# Transcriptomic and Metabolomic Analysis Reveals the Molecular Mechanisms of the Impact on the Fruiting Body Phenotype of *Lentinula edodes* Under Different Light Conditions

**DOI:** 10.3390/jof12060439

**Published:** 2026-06-16

**Authors:** Ning Jiang, Hao-Ran Dong, Hai-Long Yu, Mei-Na He, Zheng-Peng Li, Feng Zhou, Yu Li, Chang-Xia Yu, Qiao-Zhen Li

**Affiliations:** 1 Institute of Edible Fungi, Shanghai Academy of Agricultural Sciences, National Engineering Research Center of Edible Fungi, Shanghai 201403, China; jiangning@saas.sh.cn (N.J.);; 2 Shanghai Guosen Biotechnology Co., Ltd., Shanghai 201403, China

**Keywords:** *Lentinula edodes*, fruiting bodies, light condition, transcriptomic, metabolomic, molecular mechanisms

## Abstract

Light quality is a pivotal environmental signal governing the morphogenesis and metabolic programming of edible fungi. This study evaluated the effects of eight light qualities—red (R), green (G), blue (B), red-green (RG), red-blue (RB), green-blue (GB), red-green-blue (RGB), and dark control (CK)—on the agronomic traits of *Lentinula edodes*. Among all treatments, blue light (B) emerged as the most effective regulator, yielding the highest productivity per log (228.12 g), and significantly enhancing pileus diameter (45.17 mm) and stipe thickness (29.45 mm). To elucidate the underlying molecular mechanisms, integrated transcriptomic and metabolomic analyses were performed on primordia and mature fruiting bodies. Transcriptomic profiling identified 4280 differentially expressed genes (DEGs) under blue light, which were significantly enriched in energy metabolism and structural development pathways. Metabolomic analysis revealed 45 differentially expressed metabolites (DEMs), highlighting a 7.64-fold upregulation of 9(S)-HPODE and a marked downregulation of *L*-arginine at the harvest stage under blue light. Multi-omics integration demonstrated that blue light orchestrates a strategic metabolic shift: it activates arginine and proline metabolism during the primordial stage, maintains linoleic acid metabolism throughout development, and triggers alanine, aspartate, and glutamate metabolism during the transition to maturity. These pathways facilitate the conversion of amino acids into energy precursors and enhance cell membrane fluidity through unsaturated fatty acid synthesis to support rapid growth. Conversely, red light treatment triggered stress-related MAPK signaling, delayed primordium formation, and redirected resources toward stipe elongation via phenylalanine accumulation, resulting in significantly lower yields. In conclusion, this study confirms that blue light is the optimal condition for *L*. *edodes* cultivation, providing a robust molecular foundation for precision light-regulation strategies to maximize yield and quality in commercial production.

## 1. Introduction

Among tens of thousands of mushroom species identified worldwide, only approximately 20 have been successfully domesticated for commercial cultivation. A prominent example is *Lentinula edodes* (Berk.) Pegler, which is widely cultivated due to its distinctive culinary and medicinal properties [1,2,3]. As nutritionally and functionally valuable agricultural products, edible fungi exhibit growth, development, and quality traits tightly regulated by environmental factors including temperature, humidity, and light [4,5,6]. In particular, light acts not only as a critical trigger for fruiting body differentiation, but also modulates bioactive compound accumulation and morphogenesis by regulating metabolic pathways and gene expression [7,8,9,10].

Recent advances in omics technologies, including transcriptomics and metabolomics, have enabled investigations into light regulation in edible fungi across physiological and molecular levels [11,12,13,14]. Studies have demonstrated that light quality specifically influences mycelial growth, primordium differentiation, and fruiting body morphology, resulting in distinct phenotypic outcomes [15,16,17]. This regulatory process exhibits significant stage specificity, as the light requirements differ fundamentally between the mycelial growth and fruiting body development phases.

Light regulates spore germination, physiological adaptations, developmental programming, pathogenicity, circadian rhythms, stress responses, and metabolic pathways [8,18,19,20,21,22]. Although darkness generally promotes mycelial elongation in most edible fungi, fruiting body development is strictly light-dependent. For instance, primordia of *Pleurotus ostreatus* (Jacq.) P. Kumm fail to differentiate into mature sporocarps in the absence of light [23], and *L. edodes* mycelia do not form brown membranes without light exposure [24], underscoring the essential role of light in completing the fungal life cycle.

Blue light plays an irreplaceable role in promoting fruiting body differentiation, morphological improvement, and yield enhancement. In *L*. *edodes*, blue light significantly modifies the morphological characteristics of fruiting bodies: compared with the dark control, blue light induces larger pileus diameter, thicker stipe, shorter stipe length, and darker pileus color, confirming that blue light directionally modulates the development of the pileus and stipe [25,26]. In *P*. *ostreatus*, red light promotes mycelial growth, while blue light increases fruiting body diameter. Furthermore, vitamin D_2_ content in *P. ostreatus* fruiting bodies is higher under blue light and red-blue combined light [23]. At the molecular level, blue light-induced changes in *L. edodes* mainly involve differential expression of genes related to light perception, signal transduction, melanin synthesis, cell wall formation, carbohydrate metabolism, and fruiting body development [27]. In *Flammulina velutipes* (Curtis) Singer, green and blue light darken fruiting body color, whereas red light has no significant effect. Transcriptome analysis reveals the highest number of DEGs (257) under blue light. DEGs downregulated by both green and blue light are enriched in monooxygenase-related pathways. Notably, only blue light induces upregulation of the pigment-related gene *Fvpal1* and a hub gene containing a pyridoxal-dependent decarboxylase domain in the PPI network, both of which may participate in blue light-mediated pigment metabolism [28]. Collectively, these findings highlight that light exerts species-specific regulatory effects on growth, fruiting body morphology, secondary metabolite accumulation, and key functional gene expression in edible fungi such as *L. edodes*, *P. ostreatus*, and *F. velutipes*, while red and green light exert weaker or more specialized effects.

This study systematically investigates the effects of different light qualities on the yield, key agronomic traits, transcriptome, and metabolome of *L. edodes* using staged light regulation. Through multi-omics integration, we aim to elucidate the molecular mechanisms by which light governs growth, development, and quality formation in *L. edodes*, thereby providing a theoretical foundation and practical guidance for precision light-mediated cultivation.

## 2. Materials and Methods

### 2.1. Materials

Strain F2 was provided by the Shanghai Edible Fungus Sub-center of the Agricultural Microorganism Preservation Center. The PDA medium was prepared using 200 g peeled potatoes, 20 g glucose, and 20 g agar dissolved in distilled water to a final volume of 1 L, then sterilized at 121 °C for 20 min at natural pH. The cultivation substrate consisted of 86% wood chips, 13% bran, and 1% calcium carbonate (by weight), mixed with water to reach 54–56% moisture content and pH 5.8–6.2. Shade cloth with a light transmittance of 0 lux was supplied by Qingdao Lvsheng Matter Technology Co., Ltd. (Qingdao, China). LED lamps of different light qualities were purchased from Wuhan Zhongliyuan Biotechnology Co., Ltd. (Wuhan, China). Bed frames and culture room were provided by Shanghai Guosen Biotechnology Co., Ltd. (Shanghai, China). The relevant website for fungal name author abbreviations is https://www.indexfungorum.org/names/AuthorsOfFungalNames.asp accessed on 9 June 2026.

### 2.2. Fungus Log Production

Cultivation substrates were filled into polyethylene plastic bags (fold width: 15 cm, length: 33 cm) using a punching machine, with each fungus log containing (1.0 ± 0.1) kg of substrate. Fungus logs were sterilized at 121 °C for 3 h, cooled, and inoculated under aseptic conditions, then incubated in a dark room at 25 °C. After full mycelial colonization, holes were punched for aeration, and light was applied to induce brown mycelial membrane formation. Upon full maturation of mycelia and completion of browning, the outer plastic bags were removed. Adequate water supplementation was performed during the fruiting stage to compensate for water loss. The culture room was maintained at 15–20 °C with 80–90% relative humidity.

### 2.3. Cultivation Process

**Figure 1:**
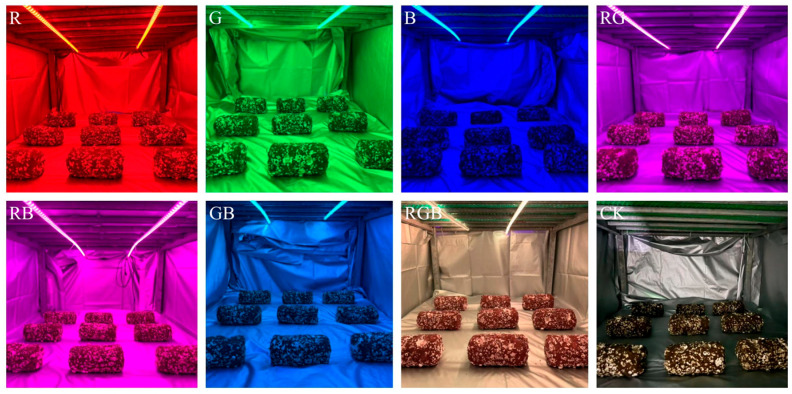
Different light illumination treatment. R, G, B, RG, RB, GB, and RGB are respectively red light, green light, blue light, red-green light, red-blue light, green-blue light, and red-green-blue light. CK denotes treatment under the dark environment.

Eight treatment groups were established: red (R), green (G), blue (B), red-green (RG), red-blue (RB), green-blue (GB), red-green-blue (RGB), and complete darkness (CK). Each group was isolated using 0 lux light-blocking cloth. Two LED lamps were installed 50 cm above the logs at a light intensity of 600 lux with 24 h daily illumination. Each treatment included nine biological replicates (Figure 1).

### 2.4. Morphological Characteristics of Fruiting Bodies

Upon reaching harvest maturity, the number of primordia and yield per log were recorded. The agronomic traits of fruiting bodies were determined by referring to the People’s Republic of China Agricultural Industry Standard NY/T 2560-2014, Guidelines for the conduct of tests for distinctness, uniformity and stability—Xianggu [29]. For each treatment, 10 fruiting bodies were randomly selected to measure the bud number, the yield of a single log, the weight of the fruiting body, the weight of the pileus, the weight of the stipe, the diameter of the pileus, the thickness of the pileus, the length of the stipe, the diameter of the stipe, the ratio of the diameter of the pileus to the length of the stipe, and the ratio of the diameter of the pileus to the diameter of the stipe.

The mature fruiting bodies of *L. edodes* were selected. The colors of the pileus and stipe of *L. edodes* were measured by a portable precision color difference meter (Hubei Tuso Technology Co., Ltd., Wuhan, China). A total of 10 fruiting bodies were selected for each treatment for measurement, and each measurement was repeated three times to obtain the average value of L*, a*, b*, and C* values.

### 2.5. Sample Preparation

According to the previous determination results of the yield and agronomic traits of *L. edodes* fruiting bodies under different light quality conditions, the *L. edodes* fruiting bodies at the primordium and harvest stage from the red light group and the blue light group were selected, taking the mycelium at the formation of the brown mycelium membrane completion stage as the control group. Samples were sent to Shanghai Personal Biotechnology Co., Ltd. (Shanghai, China) for transcriptome sequencing and metabolome analysis. There were a total of five groups, named group Z0 (control group), group ZR1 (the primordium stage under red light), group ZR2 (the harvest stage under red light), group ZB1 (the primordium stage under blue light), and group ZB2 (the harvest stage under blue light).

### 2.6. mRNA-Seq Library Construction, RNA Sequencing, and Transcriptome Analysis

Total RNA was extracted from 15 samples (three replicates per group) using the Trizol reagent kit and sequenced on the Illumina HiSeqTM 4000 platform. Raw sequencing reads were filtered and processed as previously described in Xiong [30]. The RNA-Seq clean data can be obtained at NCBI BioProject: PRJNA1405144. In this study, FPKM was used as an indicator to measure the transcript or gene expression levels. Genes with |log_2_FC| ≥ 1 and an adjusted *p* < 0.05 were regarded as differentially expressed. GO and KEGG enrichment analyses of DEGs were further conducted by employing the clusterProfiler R package.

### 2.7. Detection and Analysis of Metabolites

Metabolites were extracted from 30 samples (six replicates per group). Each 200 mg sample was mixed with 0.6 mL methanol containing 4 ppm 2-chlorophenylalanine, vortexed, ground at 60 Hz for 90 s, sonicated for 15 min, and centrifuged at 12,000 rpm for 10 min at 4 °C. The supernatant was filtered through a 0.22 μm membrane and analyzed using LC-MS. Chromatographic separation was performed on an ACQUITY UPLC^®^ HSS T3 column at 40 °C. Gradient elution used 0.1% formic acid in water and acetonitrile, or 5 mM ammonium formate in water and acetonitrile at 0.25 mL/min. Mass spectrometry was operated in positive/negative mode with a spray voltage of 3.5 kV/−2.5 kV. Full scan mass range was *m*/*z* 81–1000 at 70,000 resolution.

### 2.8. Integrated Transcriptome and Metabolome Analysis

After normalizing the transcriptomic and metabolomic data, correlation analysis and O2PLS analysis were carried out [31,32]. Using a reference standard where the Pearson correlation coefficient |r| > 0.8 and the *p* < 0.05, KEGG enrichment analysis was performed to obtain the common enrichment pathways of differential metabolites and differentially expressed genes.

### 2.9. Real-Time Quantitative PCR

The methods of cDNA synthesis and real-time quantitative PCR were consistent with those described by Zhao [33]. The expression level of *L. edodes* ribosomal protein L4 (*Rpl4*) gene was used as the internal control, with forward primers 5′-AATCGTAGACACCGTCAGCG-3′; reverse 5′-TGACGAAACGGCCAAGATGA-3′ [34]. q-PCR amplifications were performed in an optical 384-well plate. Relative expressions were calculated using the 2^−ΔΔCt^ method [35].

### 2.10. Statistical Analysis

Data are expressed as mean ± SD. Data analysis was performed followed by Duncan’s multiple range test (*p* < 0.05) and Pearson correlation, conducted with SPSS 23.0. Figures were generated using GraphPad Prism 9.

## 3. Results

### 3.1. Yield and Morphological Characteristics Under Different Light Conditions

**Figure 2:**
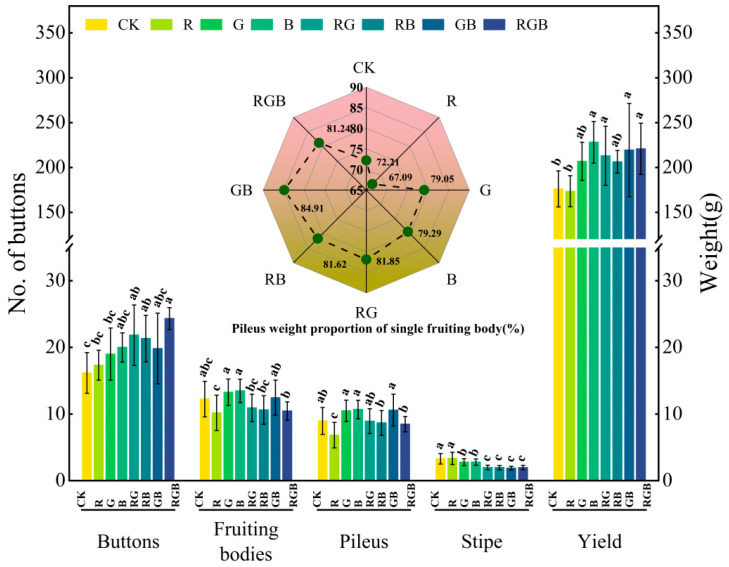
Effects of different light quality treatments on fruiting body development and yield of *L. edodes*. Radar chart showing the pileus weight proportion of a single fruiting body under different light quality treatments: CK (dark environment), R (red light), G (green light), B (blue light), RG (red-green light), RB (red-blue light), GB (green-blue light), and RGB (red-green-blue light). Bar charts representing the number of buttons, weight of fruiting bodies, weight of pileus, weight of stipe, and total yield per log under different light quality treatments, respectively. Different lowercase letters indicate significant differences at the *p* < 0.05.

**Figure 3:**
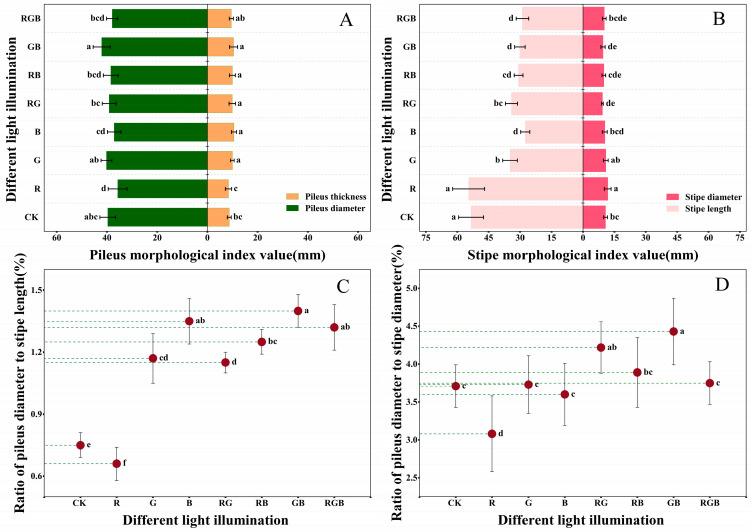
Effects of different light quality treatments on morphological characteristics of *L. edodes* fruiting bodies. (**A**) Bar chart showing the pileus diameter and pileus thickness under different light quality treatments. (**B**) Bar chart representing the stipe length and stipe diameter under different light quality treatments. (**C**) Scatter plot of the ratio of pileus diameter to stipe length under different light quality treatments. (**D**) Scatter plot of the ratio of pileus diameter to stipe diameter under different light quality treatments. Different lowercase letters indicate significant differences at the *p* < 0.05.

Under suitable cultivation conditions, *L. edodes* transitioned from formation of brown mycelium membrane completion to fruiting body maturation in about 7 days. Different light qualities have a great influence on the yield of *L. edodes* logs (Figure 2 and Figure 3). Under blue light irradiation, the yield per log is the highest (228.12 g), followed by red-green-blue light, where the yield per log is 220.82 g. Under green-blue light treatment, the yield per log is 219.40 g. Under red light and complete darkness conditions, there are few primordia; few *L. edodes* buds formed on the log, and the yield is low. The yields per log are 173.53 g and 176.03 g, respectively. When *L. edodes* fruiting bodies were exposed to eight distinct light quality treatments, significant differences were detected in various agronomic traits. Under blue light and green light treatments, the single fruiting body weight exceeded 13 g, and the proportion of pileus exceeded 67.09%, accompanied by a high weight ratio of the pileus and a well-formed fruiting body shape. Under green-blue mixed light and dark conditions, the single fruiting body weight was over 12 g. In contrast, under red light and dark conditions, the stipe displayed a longer length and greater biomass, but the overall fruiting body shape was poor. In conclusion, the agronomic traits of *L. edodes* fruiting bodies are optimized when irradiated with blue light or green light.

**Figure 4:**
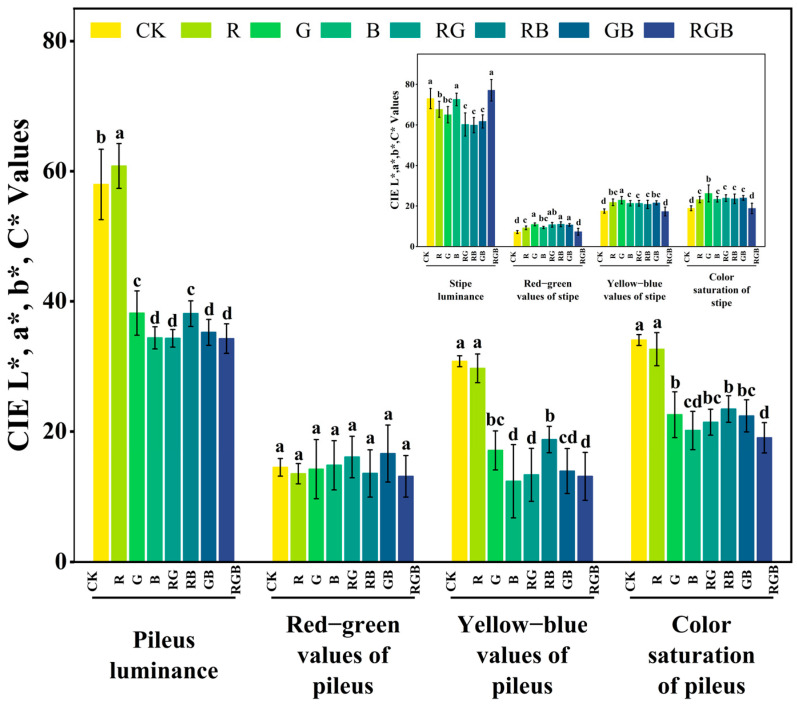
Effects of different light quality treatments on L*, a*, b*, and C* color parameters of *L. edodes* pileus and stipe. Bar charts representing lightness (L*), red-green coordinate (a*), yellow-blue coordinate (b*), and chroma (C*) values of *L. edodes* pileus under different light quality treatments. The inset shows the corresponding color parameters of the stipe under the same treatments. Different lowercase letters indicate significant differences at the *p* < 0.05.

The color parameters of the pileus and stipe in *L. edodes* fruiting bodies were determined using a color difference meter (Figure 4). Among the measured parameters, L* denotes lightness, a* represents the red-green axis, b* indicates the yellow-blue axis, and C* stands for saturation. Under red light irradiation and dark conditions, the L* values of both the pileus and stipe in *L. edodes* fruiting bodies were relatively high, presenting a whitish appearance. Across different light quality treatments, no significant difference was observed in the a* value of the pileus. The b* value of the pileus was consistently positive, indicating a dominant yellow hue. Specifically, the pileus exhibited higher b* and C* values under red light and dark conditions. For the stipe, the highest b* and C* values were recorded under the red-green-blue mixed light treatment, while the lowest values were found under red light treatment. These results collectively indicate that light quality significantly modulates the color characteristics of *L. edodes* fruiting bodies, with red light and dark conditions fading the colors of the pileus and stipes.

### 3.2. RNA Sequencing and Transcriptome Assembly

A total of 15 cDNA libraries were constructed and sequenced using the Illumina platform. After raw read cleaning and quality filtering, an average of 41,991,626 clean reads per sample was obtained, with the Q30 ≥ 92.38%. When mapping clean reads to the *L. edodes* reference genome, the mapping rate ranged from 89.97% to 92.82%, and more than 88.43% of the mapped reads were uniquely aligned to the genome. The sequencing saturation curve demonstrated that the sequencing depth was sufficient to capture the majority of expressed genes in the samples. Furthermore, the sequencing data exhibited high quality, and strong correlations were observed among biological replicates; these characteristics confirmed the reliability of the data for subsequent analyses. Differentially expressed genes (DEGs) were identified under the criteria of *p*-adjust < 0.05 and |log_2_FoldChange| ≥ 1.

**Figure 5:**
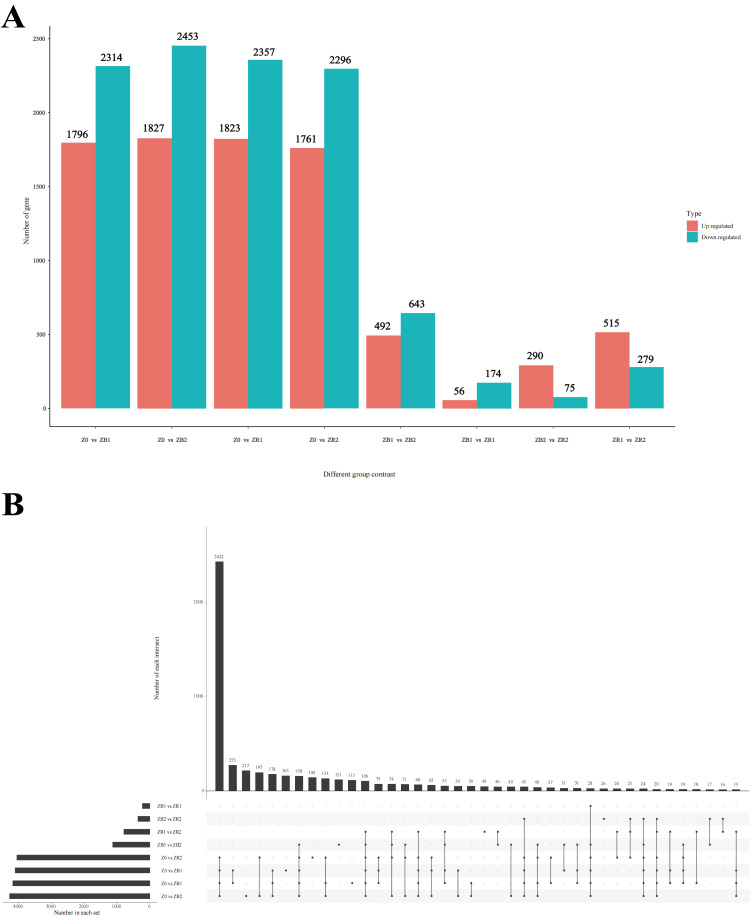
Differentially expressed genes (DEGs) in *L. edodes* under different light quality treatments and developmental stages. (**A**) Bar chart displaying the number of upregulated and downregulated DEGs identified in pairwise comparisons across different light quality treatments and developmental stages: Z0, ZB1, ZB2, ZR1, and ZR2. (**B**) UpSet plot illustrating the distribution of overlapping and unique DEGs across all comparison groups. A single point on the *x*-axis represents the number of DEGs unique to that comparison group, and connected points on the *x*-axis represent the number of DEGs shared among the connected comparison groups.

The results revealed variations in both the types and expression levels of genes in *L. edodes* fruiting bodies under different light quality treatments. Specifically, light exerted a regulatory effect on the expression of genes associated with the fruiting body development stage (Figure 5). Among all comparison groups, the number of DEGs between the blue-light-treated fruiting body stage and blue-light-treated mycelium stage was the highest (4280), with 1827 upregulated and 2453 downregulated. In contrast, the lowest number of DEGs was detected between the red-light-treated primordium stage and blue-light-treated primordium stage, with only 230 DEGs (56 upregulated and 174 downregulated genes). Notably, regardless of whether the samples were treated with blue light or red light, the number of DEGs among the primordium stage, fruiting body stage, and mycelium at the formation of brown mycelium membrane completion stage was substantially higher than that between the fruiting body stage and primordium stage (intra-light-quality developmental transition comparison). Additionally, all comparison groups exhibited group-specific DEGs, except for the f red light primordium stage vs. blue light primordium stage pair, which had no unique DEGs. Subsequently, functional enrichment analyses, including Gene Ontology (GO) annotation and Kyoto Encyclopedia of Genes and Genomes (KEGG) metabolic pathway analysis, were performed on all the aforementioned DEGs to explore their potential biological functions and associated metabolic pathways.

**Figure 6:**
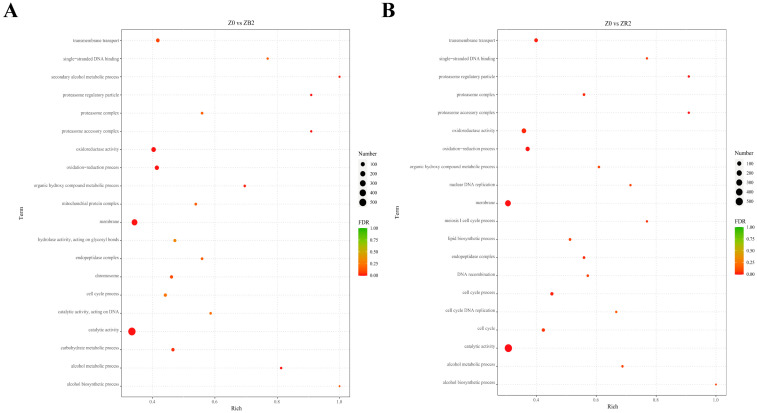
Gene Ontology (GO) enrichment analysis of DEGs in *L. edodes* under different light quality treatments and developmental stages. (**A**) GO enrichment of DEGs between Z0 and ZB2. (**B**) GO enrichment of DEGs between Z0 and ZR2. The *x*-axis represents the Rich factor, the *y*-axis shows the GO term description, the size of each dot indicates the number of DEGs annotated to that term, and the color gradient represents the FDR value.

GO enrichment analysis indicated that DEGs under blue and red light treatments were primarily concentrated in the biological process category, with the number of upregulated genes exceeding that of downregulated genes. For visualization, the top 20 most significantly enriched GO term entries were selected for display (Figure 6 and Figure S1). Under blue light exposure, the most significantly enriched GO terms included: ribosome, carbohydrate metabolic process, secondary alcohol metabolic process, mitochondrial protein complex, catalytic activity, acting on DNA, hydrolase activity, and acting on glycosyl bonds. Under red light, the most significantly enriched GO terms included: mitochondrial protein complex, organic hydroxy compound metabolic process, DNA recombination, lipid biosynthetic process, homologous recombination, and terpene synthase activity.

**Figure 7:**
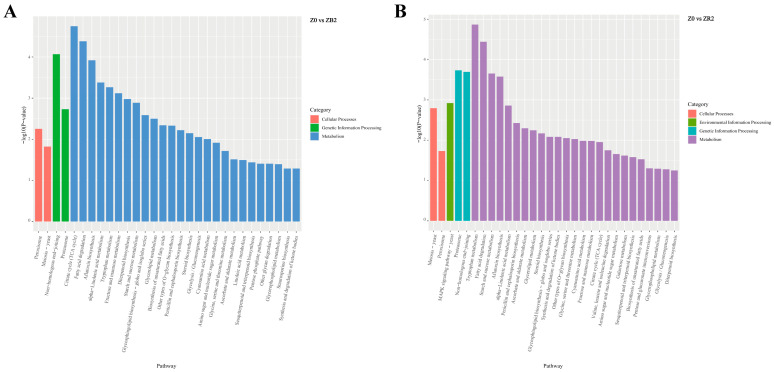
KEGG pathway enrichment analysis of DEGs in *L. edodes* under different light quality treatments and developmental stages. (**A**) KEGG enrichment of DEGs between Z0 and ZB2. (**B**) KEGG enrichment of DEGs between Z0 and ZR2. Bar charts display the top 30 most significantly enriched KEGG pathways from pairwise comparisons across different light quality treatments and developmental stages. The *y*-axis represents the KEGG pathway name, the *x*-axis shows the negative logarithm of the *p*-value, with higher values indicating more significant enrichment.

For KEGG enrichment analysis of DEGs, the top 30 pathways with the smallest *p*-values were selected for visualization, as presented in Figure 7 and Figure S2. Under blue light treatment, DEGs were predominantly enriched in the citrate cycle (TCA cycle) and glycolysis pathways. Specifically, in the comparison between the control group (Z0) and the blue-light-treated group B2 (Z0 vs. ZB2), 19 genes involved in the TCA cycle metabolic pathway were significantly upregulated (*p* < 0.001). From the inoculation stage to the harvest stage, the core carbon metabolism pathway remained continuously activated, which provided sufficient energy for the development of *L. edodes* fruiting bodies. Under red light treatment, the MAPK signaling pathway was significantly activated. In the comparison between the control group (Z0) and the red-light-treated group R1 (Z0 vs. ZR1), 30 genes in this pathway were upregulated and 36 were downregulated (*p* < 0.05); in the comparison between the control group (Z0) and the red light-treated group R2 (Z0 vs. ZR2), 36 genes were upregulated and 42 were downregulated (*p* < 0.05). These results suggest that red light may induce stress responses in *L. edodes* mycelia and regulate cell growth processes.

### 3.3. Metabolomic Analysis

**Figure 8:**
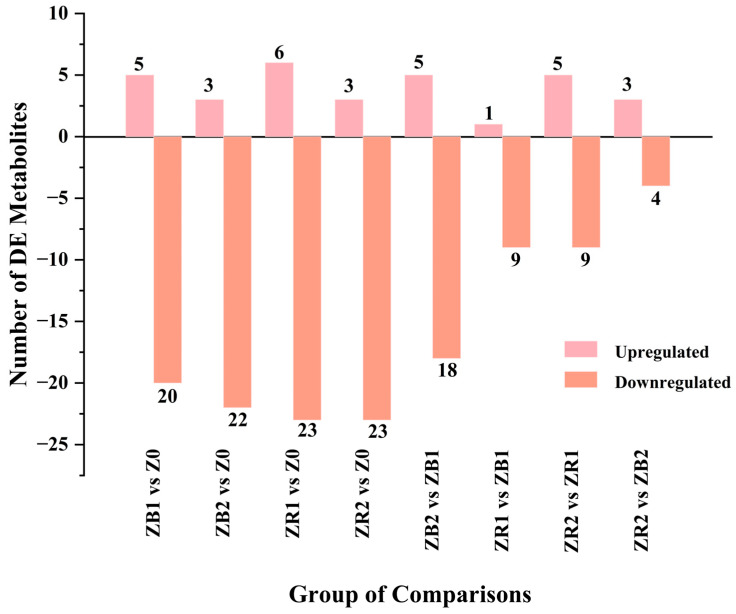
Number of DEMs in *L. edodes* under different light quality treatments. Bar chart shows the number of upregulated and downregulated DEMs identified in pairwise comparisons across different light quality treatments and developmental stages.

Differentially expressed metabolites (DEMs) were screened based on the identification criterion of variable importance in projection (VIP) ≥ 1 and *p* ≤ 0.05. As shown in the column chart, a total of 45 DEMs were identified across all comparison groups, with the number of downregulated metabolites exceeding that of upregulated ones (Figure 8). Diverse DEMs were detected among different light treatment groups, covering multiple metabolic categories. These metabolites span multiple categories, including amino acids like *L*-glutamic acid and *L*-phenylalanine, nucleotides such as UMP and GMP, fatty acids such as α-linolenic acid and 9(S)-HPODE, and hormone-related compounds like 17 α-estradiol and prostaglandin H2.

Upon blue light treatment, 9(S)-HPODE exhibited a significant upregulation. Specifically, its level in the ZB2 stage was 7.64-fold higher than that in the Z0 stage. This significant increase suggests that 9(S)-HPODE may be actively involved in blue light-induced lipid peroxidation processes or play a crucial role in blue light-mediated signaling pathways, both of which could be linked to blue light’s regulatory effects on *L. edodes* fruiting body development. Concurrently, *L*-arginine was markedly downregulated under blue light treatment. This downregulation potentially leads to the inhibition of nitrogen metabolism-related pathways, thereby affecting the overall physiological and metabolic activities of the fungus.

**Figure 9:**
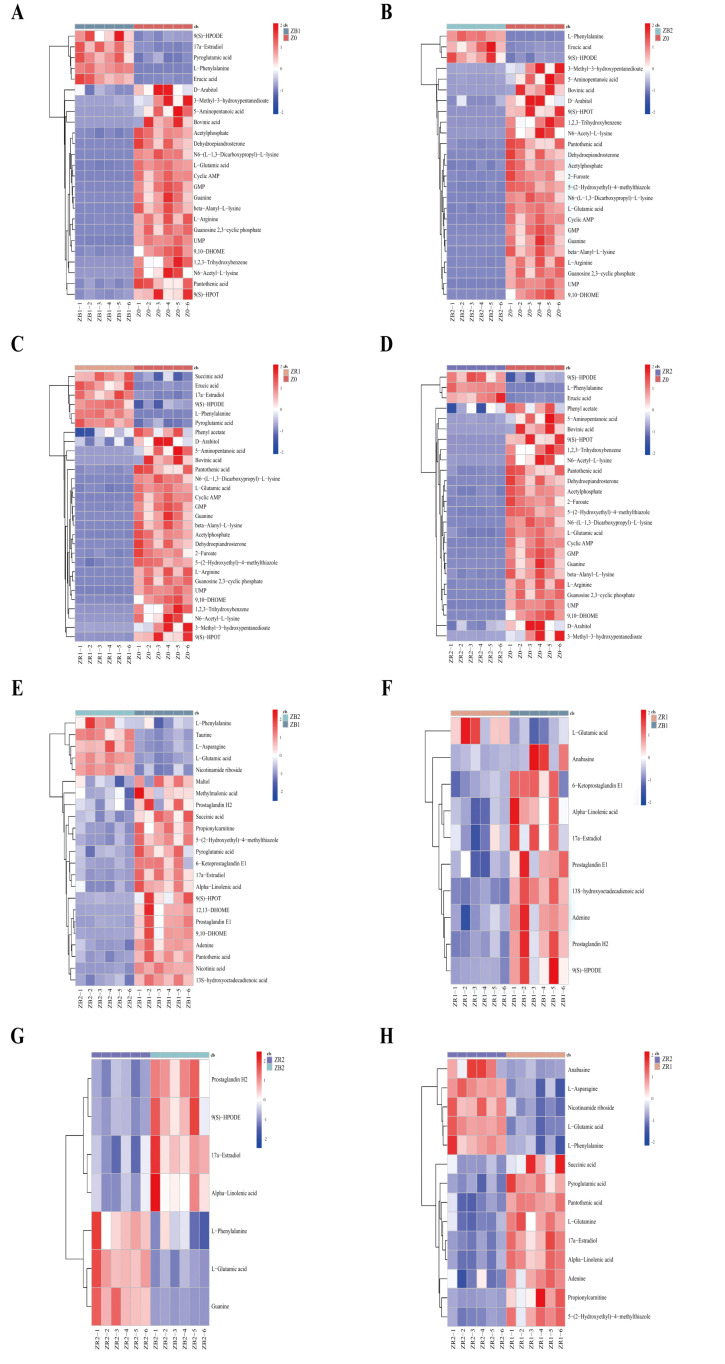
Heatmaps of DEMs in *L. edodes* under blue and red light treatments. (**A**) Z0 vs. ZB1. (**B**) Z0 vs. ZB2. (**C**) Z0 vs. ZR1. (**D**) Z0 vs. ZR2. (**E**) ZB1 vs. ZB2. (**F**) ZB1 vs. ZR1. (**G**) ZB2 vs. ZR2. (**H**) ZR1 vs. ZR. Heatmaps show the expression patterns of DEMs identified in pairwise comparisons across different light quality treatments and developmental stages. The relative content of metabolites is displayed by different colors, with columns representing samples and rows representing metabolites. Color gradients from blue to red represent the downregulation to upregulation of metabolites.

In contrast (Figure 9), under red light treatment, ZR2 vs. Z0 comparison revealed that *L*-phenylalanine had an exceptionally high fold change FC (94.8). Such a substantial increase implies that *L*-phenylalanine might contribute to stipe growth by facilitating cell elongation or enhancing protein synthesis, both of which are essential for the development of the stipe. Moreover, the dehydroepiandrosterone in the ZR2 vs. Z0 comparison showed an extremely low FC (6.7 × 10^−9^). This significant decrease indicates that red light may inhibit steroid metabolism.

### 3.4. Comprehensive Analysis of the Transcriptome and Metabolome

**Figure 10:**
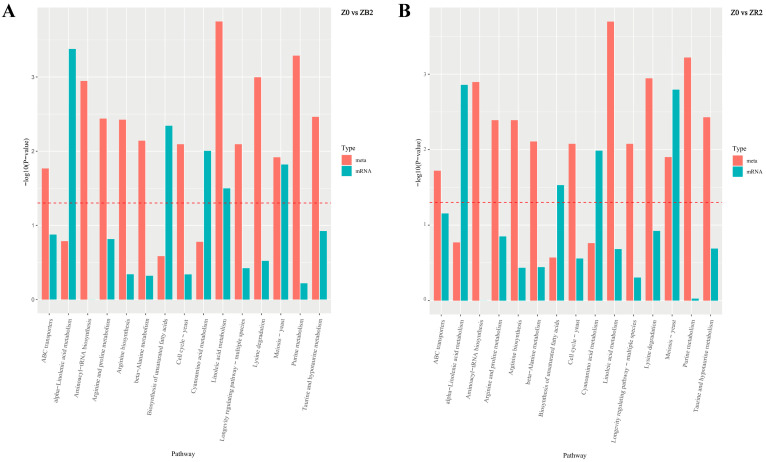
KEGG pathway enrichment analysis of integrated transcriptome and metabolome in *L. edodes* under blue and red light treatments. (**A**) Z0 vs. ZB2. (**B**) Z0 vs. ZR2. Bar charts show the enriched KEGG pathways from the correlation analysis of DEGs and DEMs across pairwise comparisons of *L. edodes* under blue light (ZB), red light (ZR), and dark control (Z0) conditions at primordium and harvest stages. In each panel, the x-axis represents the names of KEGG metabolic pathways, the *y*-axis represents the −log_10_ (*p*-value) of enrichment analysis for the two omics, and different colors denote distinct omics types. The red dashed line denotes the significance threshold of *p* = 0.05; bars above this line indicate pathways with *p* < 0.05, representing statistically significant enrichment.

To explore the regulatory relationship between DEGs and DEMs, we performed a correlation analysis followed by common KEGG pathway enrichment to characterize their interactive patterns (Figure 10 and Figure S3). At stage ZB1 vs. stage Z0, significant enrichments were observed in arginine and proline metabolism, D-arginine and D-ornithine metabolism, linoleic acid metabolism, and yeast meiosis. Stage ZB2 vs. stage Z0 retained enrichments in linoleic acid metabolism and yeast meiosis, while stage ZB2 vs. stage ZB1 highlighted alanine, aspartate and glutamate metabolism, α-linolenic acid metabolism, propanoate metabolism, and taurine and hypotaurine metabolism. For red light treatments, linoleic acid metabolism was solely enriched at stage ZR1 vs. stage Z0, with lower activation intensity than blue light, potentially contributing to delayed primordium formation and reduced yield. Stage ZR2 vs. stage Z0 showed specific enrichment in yeast meiosis, with activation timing later than blue light, putatively prioritizing stipe development. Compared to stage ZB2, stage ZR2 uniquely featured butanoate metabolism, while stage ZR2 vs. stage ZR1 emphasized alanine, aspartate and glutamate metabolism, and taurine and hypotaurine metabolism. Under blue light treatment, arginine, proline, ornithine metabolism, and linoleic acid metabolism were significantly enriched during the mushroom bud stage. During the harvesting stage, alanine, aspartate, glutamate metabolism, α-linolenic acid metabolism, and propanoate metabolism were significantly enriched, indicating that blue light may promote the conversion of amino acids into energy precursors (such as α-ketoglutarate) and enhance cell membrane fluidity through unsaturated fatty acid metabolism to support the rapid growth of fruiting bodies. Under red light treatment, only linoleic acid metabolism was enriched during the mushroom bud stage, with a lower activation degree than the blue light pathway, which may lead to slower *L. edodes* bud formation and lower yield under red light. The activation of yeast meiosis during the harvesting stage was later than that under blue light, which may mainly act on the stipe.

### 3.5. qPCR Analysis

**Figure 11:**
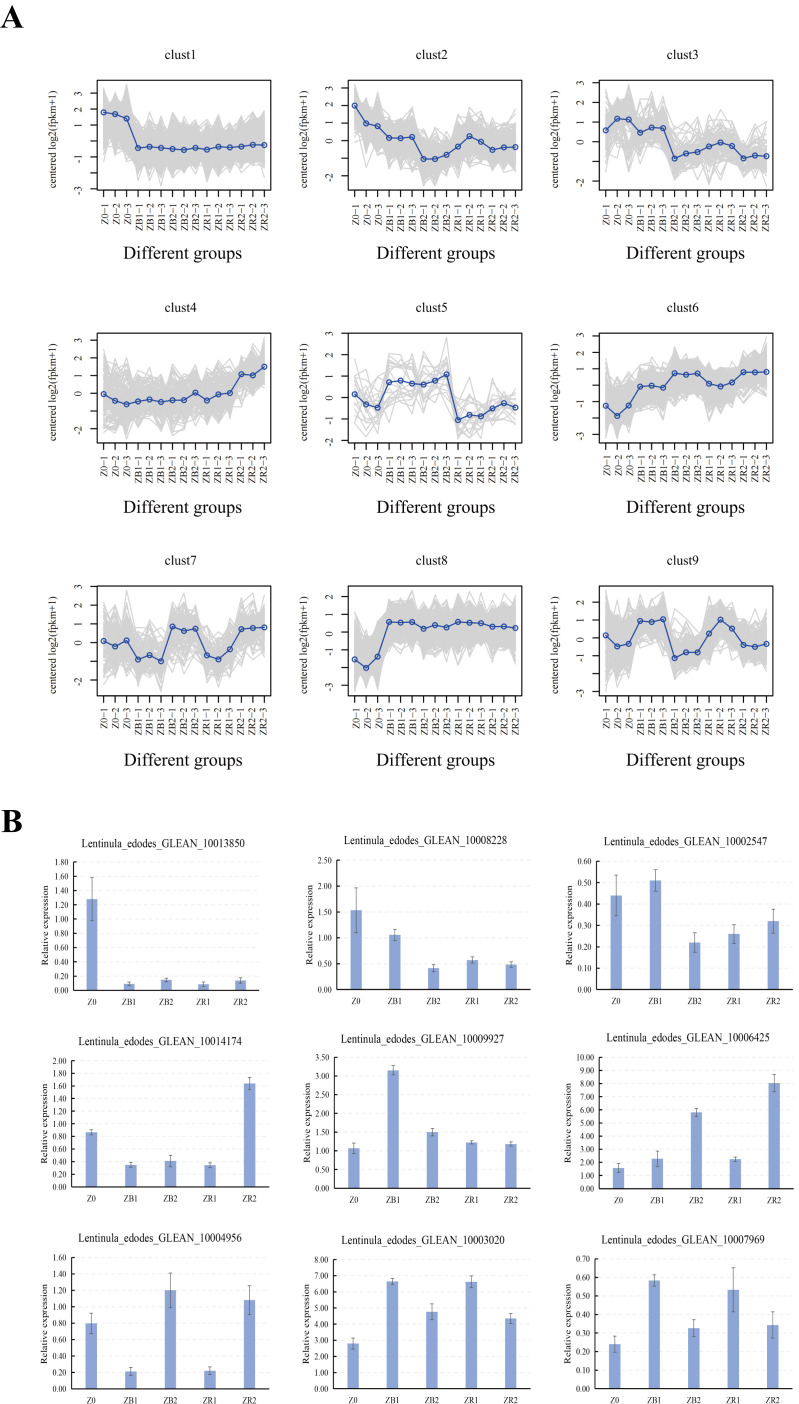
Gene expression clustering and qRT-PCR validation of DEGs in *L. edodes* under different light treatments. (**A**) Gene expression clustering profiles. Line charts showing nine distinct expression profiles of differentially expressed genes clustered from five sample groups (Z0, ZB1, ZB2, ZR1, ZR2) based on their gene expression patterns. The *x*-axis represents different sample groups, and the *y*-axis represents the centered log_2_ (fold change) of gene expression. (**B**) qRT-PCR validation of RNA-seq data. Bar charts illustrating the relative expression levels of nine selected genes measured by quantitative real-time polymerase chain reaction. The x-axis indicates the five sample groups (Z0, ZB1, ZB2, ZR1, ZR2), and the *y*-axis represents the relative expression level of each gene.

Based on gene expression patterns, DEGs of five sample groups were clustered in nine profiles (Figure 11). To validate the expression data obtained by RNA sequencing, one gene from each gene expression pattern was randomly selected for qRT-PCR validation. The expression of these genes by qRT-PCR exhibited an expression profile similar to those gained by RNA-seq. The results of this analysis revealed a strong correlation between the RNA-seq and qRT-PCR data, suggesting that reliable expression results were generated via RNA-seq.

## 4. Discussion

This study presents a comprehensive investigation into the regulatory effects of different light qualities on the yield, agronomic traits, and molecular responses of *L*. *edodes*. Our findings demonstrate that blue light is the most favorable treatment, as it significantly enhances yield and improves key morphological traits of fruiting bodies. Integrated transcriptomic and metabolomic analyses reveal that these phenotypic advantages are underpinned by coordinated changes in gene expression and metabolite accumulation, primarily involving energy metabolism, amino acid conversion, and fatty acid metabolism.

Blue light treatment yielded the highest production per log (228.12 g), significantly outperforming red light and dark conditions. This is consistent with the findings of Kim [36], who reported that the fruiting body yield of both *L. edodes* cultivars was increased under blue light irradiation. Similar results have been observed in other edible fungi. In *F. velutipes*, blue light was found to enhance biomass production and growth rate, promote primordium formation, and accelerate fruiting body development [37]. Similarly, in *Pleurotus ostreatus* and *Pleurotus eryngii* (DC.) Quél., blue light treatment increased the diameter of fruiting bodies [23,38]. The superior performance under blue light can be attributed to its efficient activation of energy metabolic pathways. Our transcriptome data revealed significant enrichment in the citrate cycle (TCA cycle) and glycolysis pathways under blue light, with 19 TCA cycle genes being significantly upregulated during the harvesting stage (ZB2 vs. Z0). This suggests that blue light enhances respiratory energy production, providing ample ATP and metabolic precursors for rapid fruiting body growth and development. This finding is consistent with research on *Pleurotus ostreatus* and *Pleurotus eryngii*, where blue light was shown to upregulate genes in glycolysis and the pentose phosphate pathway to support pileus growth [13,31].

The metabolomic analysis identified 45 differentially expressed metabolites (DEMs). Under blue light, two key DEMs were notable: 9(S)-HPODE was upregulated by 7.64-fold, while *L*-arginine was downregulated. 9(S)-HPODE is a fatty acid hydroperoxide involved in lipid peroxidation and may act as a signaling molecule [39]. Its accumulation suggests that blue light may enhance lipid metabolism and oxidative signaling, potentially influencing membrane fluidity and structural development. The downregulation of *L*-arginine indicates a potential shift in nitrogen metabolism, possibly redirecting resources towards other growth-related processes.

Comprehensive metabolic pathway analysis further revealed the dynamic evolution law of metabolism regulated by blue light: starting from the primordium stage (ZB1), where arginine and proline metabolism are the core, the fungi gradually transitions to the fruiting body harvesting stage (ZB2 vs. ZB1), dominated by alanine, aspartate, and glutamate metabolism. This transition marks a strategic shift in metabolic pathways—blue light can promote the conversion of amino acids into energy precursors, thereby accurately matching the high energy demand during the development of fruiting bodies. Additionally, the sustained enrichment of linoleic acid metabolism across stages suggests enhanced biosynthesis of unsaturated fatty acids, which can improve cell membrane fluidity and integrity. In *Lentinus crinitus* (L.) Fr., blue light can induce changes in the fatty acid metabolism of mycelia, leading to an increased proportion of polyunsaturated fatty acids. Among these PUFAs, the content of linoleic acid is significantly higher than that in the dark group as well as in the red and green light groups; the increased proportion of linoleic acid may promote the fluidity of cell membranes [40].

Our results on the efficacy of blue light are consistent with studies across various mushroom species. For example, in *Flammulina filiformis* (Z.W. Ge, X.B. Liu and Zhu L. Yang) P.M. Wang, Y.C. Dai, E. Horak and Zhu L. Yang, blue light significantly increased the content of lysine in the stipe by downregulating its degradation genes [9]. In *P. eryngii*, blue light upregulated genes encoding CAZymes, which are crucial for substrate degradation and primordium differentiation [6]. This conserved role of blue light in promoting growth and quality across species highlights its fundamental importance in fungal photomorphogenesis. However, the specific metabolic pathways regulated by blue light may vary among different species: in *F. filiformis*, the core role of blue light is to significantly promote lysine accumulation, which is also a key target for its nutritional improvement. However, in the exploration of *L. edodes* in this study, the regulatory focus of blue light lies in the comprehensive reconstruction of amino acid metabolism and energy metabolism. In contrast to blue light, red light treatment resulted in lower yield and delayed primordium formation. Transcriptome analysis indicated that red light significantly activated the MAPK signaling pathway—a pathway often associated with stress responses in fungi. For instance, in *L. edodes*, red light treatment led to the up- and downregulation of numerous genes in the MAPK pathway, suggesting it may induce a stress state that diverts resources from growth to defense, thereby impairing primordia differentiation and yield.

Metabolomically, red light led to an enormous accumulation of *L*-phenylalanine (FC = 94.8) and a sharp reduction in dehydroepiandrosterone. The accumulation of phenylalanine, a precursor for lignin and flavonoid biosynthesis, might contribute to reinforced cell walls and explain the observed longer, thicker stipes under red light. However, this likely occurs at the expense of pileus development and overall yield. The severe suppression of dehydroepiandrosterone, a steroid precursor, suggests red light inhibits steroid metabolism, which could impact membrane integrity and developmental processes. The pathway analysis for red light showed minimal activation at the primordium stage and a late activation of yeast meiosis pathways at the harvesting stage. This delayed and distinct transcriptional response indicates that red light’s primary effect might be on later developmental stages, potentially promoting stipe elongation and maturation rather than the early, explosive growth required for high yield, similar to findings in *P. ostreatus* where red light promoted mycelial growth but was less effective than blue light for fruiting body induction [23].

While this study elucidates the profound impact of light quality on *L. edodes*, some limitations remain. The study focused on two key light treatments (blue and red) based on phenotypic results; future work could include a more detailed time-course analysis of other light qualities to capture dynamic transcriptional and metabolic shifts. Furthermore, the functional validation of key candidate genes (e.g., those involved in the MAPK pathway under red light or rate-limiting enzymes in the TCA cycle under blue light) through gene knockout or overexpression is essential to confirm their causal roles in the observed phenotypes.

## 5. Conclusions

In conclusion, our multi-omics analysis demonstrates that blue light serves as the most effective signal for enhancing the yield and quality of *L. edodes* by coordinately upregulating energy metabolic pathways (glycolysis, TCA cycle), modulating amino acid metabolism to supply energy precursors, and enhancing membrane fluidity through unsaturated fatty acid synthesis. In contrast, red light appears to trigger stress responses, delay primordium formation, and redirect metabolism towards stipe development, resulting in lower yields. These findings provide a robust molecular foundation for applying precise light-regulation strategies in the commercial production of *L. edodes* to optimize yield and nutritional quality.

## Data Availability

Datasets used or analyzed during the current study are available in the manuscript.

## Supplementary Materials

The following supporting information can be downloaded at: https://www.mdpi.com/article/10.3390/jof12060439/s1.
