# Supplementary material for: Transcriptomic and Metabolomic Analysis Reveals the Molecular Mechanisms of the Impact on the Fruiting Body Phenotype of Lentinula edodes Under Different Light Conditions
Source: J Fungi (Basel). 2026 Jun 16;12(6):439. doi: 10.3390/jof12060439 (PMC13302338; doi:10.3390/jof12060439)
Supplement: Supplementary file 1 [file jof-12-00439-s001.zip › jof-4341462-supplementary.pdf]

Supplementary materials

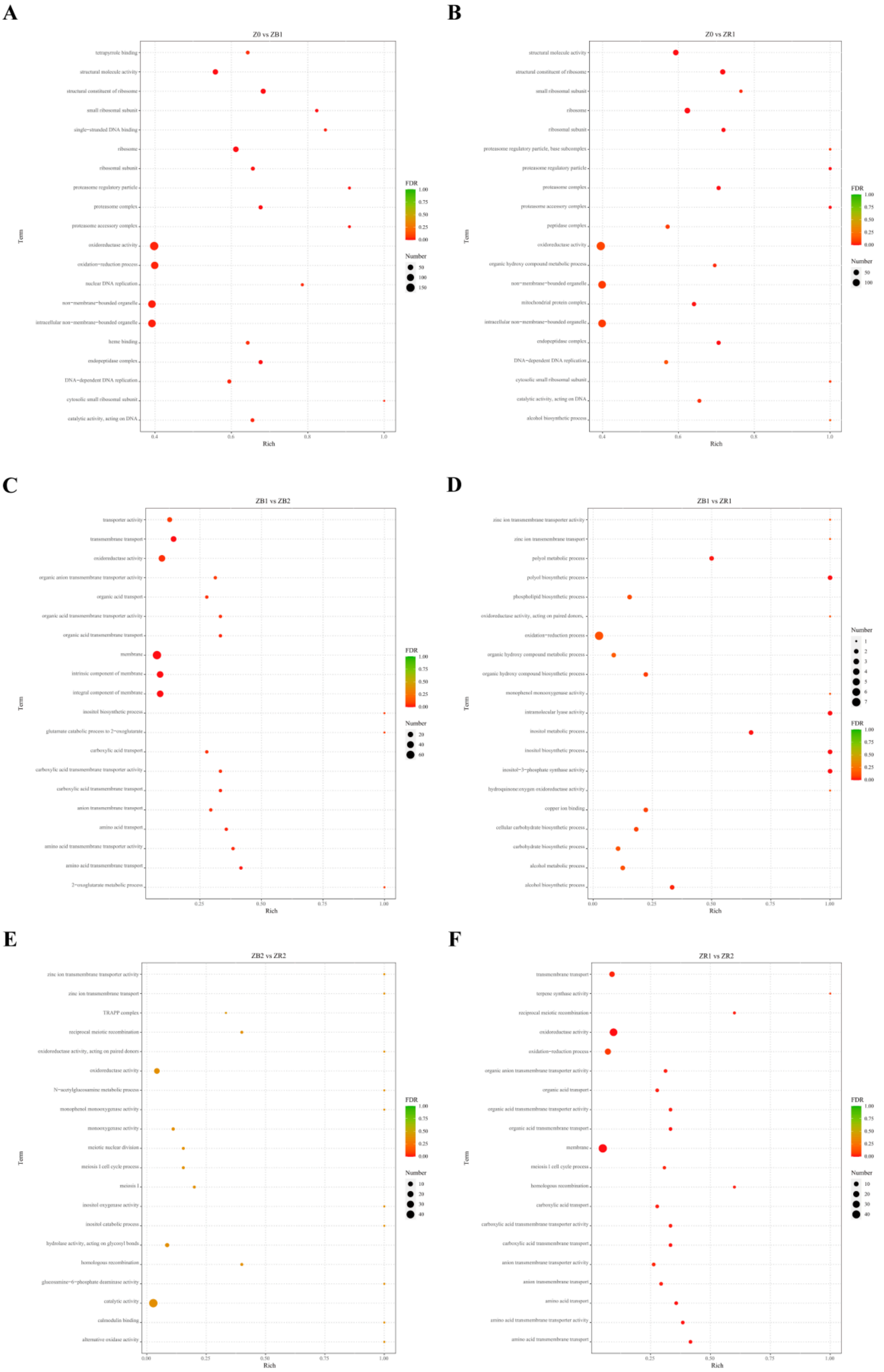

Figure S1. Gene Ontology (GO) enrichment analysis of DEGs in *L. edodes* under different light quality treatments and developmental stages. The x-axis represents the Rich factor, the y-axis shows the GO term description, the size of each dot indicates the number of DEGs annotated to that term, and the color gradient represents the FDR value.

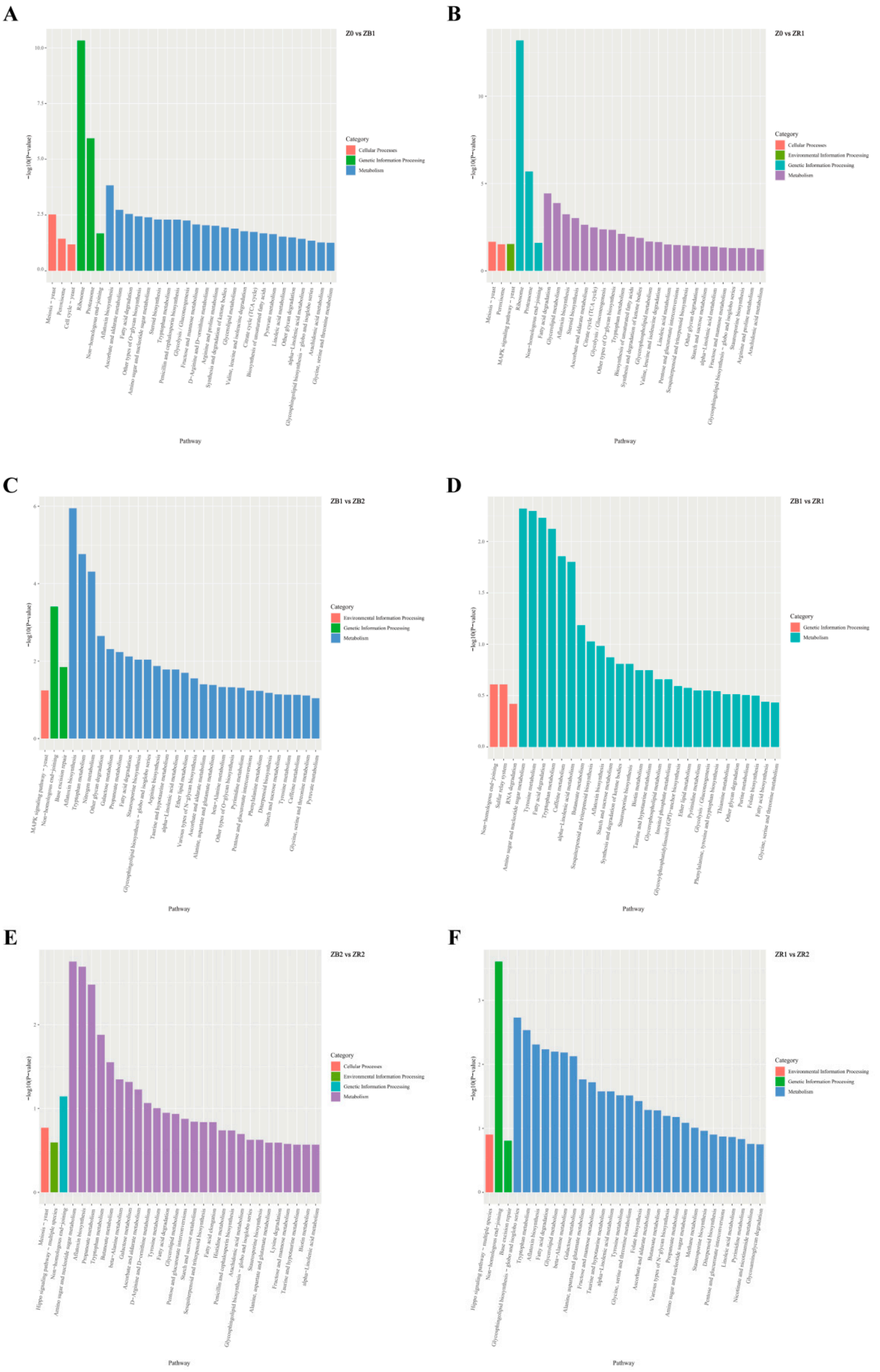

Figure S2. KEGG pathway enrichment analysis of DEGs in *L. edodes* under different light quality treatments and developmental stages. Bar charts displaying the top 30 most significantly enriched KEGG pathways from pairwise comparisons across different light quality treatments and developmental stages. The y-axis represents the KEGG pathway name, the x-axis shows the negative logarithm of the p-value, with higher values indicating more significant enrichment.

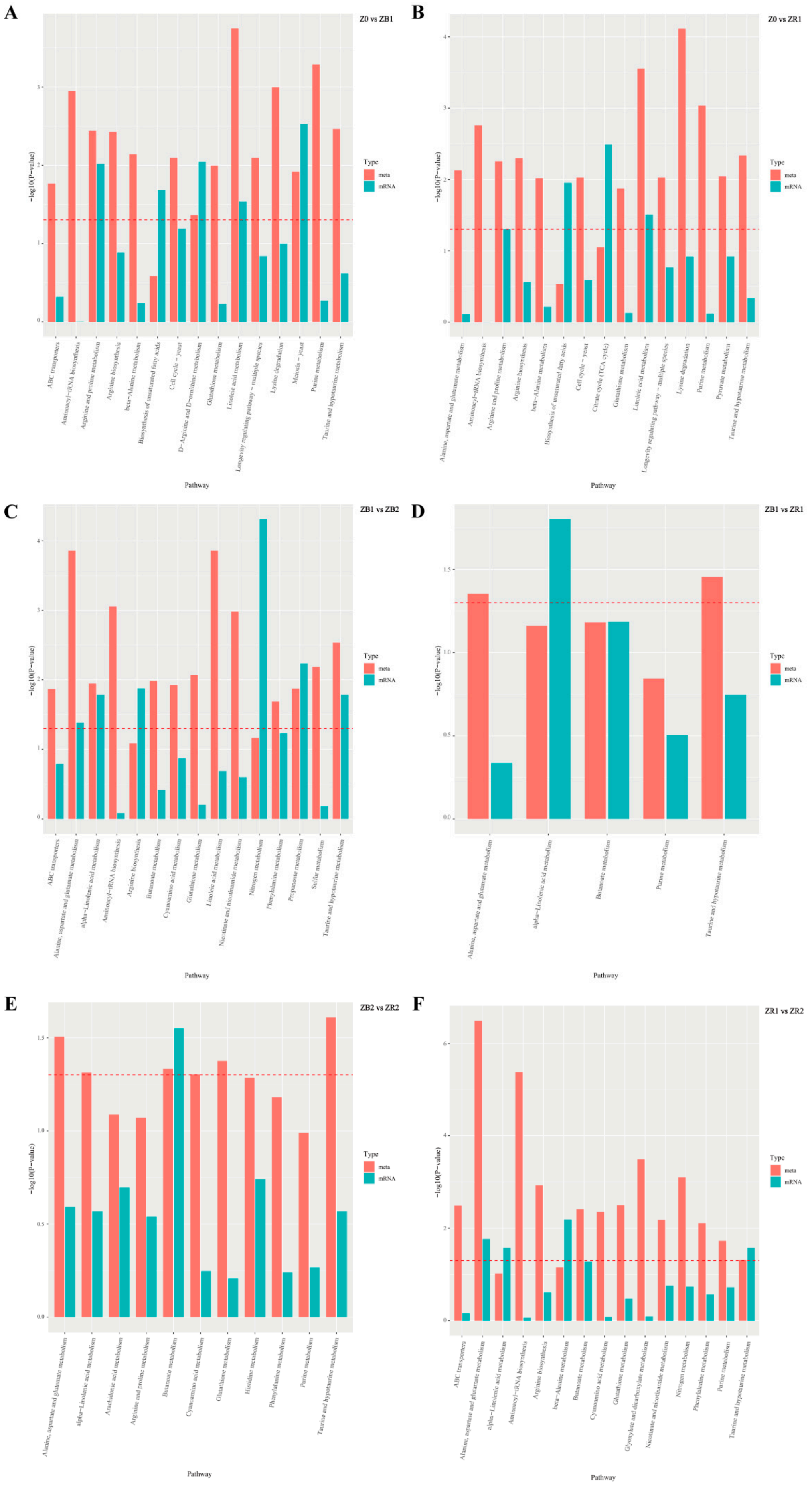

A
Z0 vs ZB1
−log10(P−value)
Type
meta
mRNA
ABC transporters
Aminoacyl−tRNA biosynthesis
Arginine and proline metabolism
Arginine biosynthesis
beta−Alanine metabolism
Biosynthesis of unsaturated fatty acids
Cell cycle − yeast
D−Arginine and D−ornithine metabolism
Glutathione metabolism
Linoleic acid metabolism
Longevity regulating pathway − multiple species
Lysine degradation
Meiosis − yeast
Purine metabolism
Taurine and hypotaurine metabolism
Pathway
B
Z0 vs ZR1
Alanine, aspartate and glutamate metabolism
Aminoacyl−tRNA biosynthesis
Arginine and proline metabolism
Arginine biosynthesis
beta−Alanine metabolism
Biosynthesis of unsaturated fatty acids
Cell cycle − yeast
Citrate cycle (TCA cycle)
Glutathione metabolism
Linoleic acid metabolism
Longevity regulating pathway − multiple species
Lysine degradation
Purine metabolism
Pyruvate metabolism
Taurine and hypotaurine metabolism
C
ZB1 vs ZB2
ABC transporters
Alanine, aspartate and glutamate metabolism
alpha−Linolenic acid metabolism
Aminoacyl−tRNA biosynthesis
Arginine biosynthesis
Butanoate metabolism
Cyanoamino acid metabolism
Glutathione metabolism
Linoleic acid metabolism
Nicotinate and nicotinamide metabolism
Nitrogen metabolism
Phenylalanine metabolism
Propanoate metabolism
Sulfur metabolism
Taurine and hypotaurine metabolism
D
ZB1 vs ZR1
Alanine, aspartate and glutamate metabolism
alpha−Linolenic acid metabolism
Butanoate metabolism
Purine metabolism
Taurine and hypotaurine metabolism
E
ZB2 vs ZR2
Alanine, aspartate and glutamate metabolism
alpha−Linolenic acid metabolism
Arachidonic acid metabolism
Arginine and proline metabolism
Butanoate metabolism
Cyanoamino acid metabolism
Glutathione metabolism
Histidine metabolism
Phenylalanine metabolism
Purine metabolism
Taurine and hypotaurine metabolism
F
ZR1 vs ZR2
ABC transporters
Alanine, aspartate and glutamate metabolism
alpha−Linolenic acid metabolism
Aminoacyl−tRNA biosynthesis
Arginine biosynthesis
beta−Alanine metabolism
Butanoate metabolism
Cyanoamino acid metabolism
Glutathione metabolism
Glyoxylate and dicarboxylate metabolism
Nicotinate and nicotinamide metabolism
Nitrogen metabolism
Phenylalanine metabolism
Purine metabolism
Taurine and hypotaurine metabolism

Figure S3. KEGG pathway enrichment analysis of integrated transcriptome and metabolome in *L. edodes* under blue and red light treatments. Bar charts showing the enriched KEGG pathways from the correlation analysis of DEGs and DEMs across pairwise comparisons of *L. edodes* under blue light (ZB), red light (ZR) and dark control (Z0) conditions at primordium and harvest stages. In each panel, the x-axis represents the names of KEGG metabolic pathways, the y-axis represents the $-\log_{10}$ (p-value) of enrichment analysis for the two omics, and different colors denote distinct omics types.
